# Platelet Satellitism Causing Apparent Loss of Response to Eltrombopag in a Patient With Immune Thrombocytopenia

**DOI:** 10.1111/ijlh.70071

**Published:** 2026-02-16

**Authors:** Torsten Ebeling, Stefanie Huber, Leah Bernadette Klingel, Carlo Zaninetti

**Affiliations:** ^1^ Department of Pediatric Oncology and Hematology University Medicine Greifswald Greifswald Germany; ^2^ Department of Transfusion Medicine University Medicine Greifswald Greifswald Germany

A 12‐year‐old patient was diagnosed with immune thrombocytopenia. Following an inadequate response to corticosteroids, intravenous immunoglobulin and romiplostim, treatment was switched to eltrombopag, which led to stabilization of platelet counts. After 4 months of stable platelet counts above 100x10^3^/μL, an automated complete blood count (CBC) performed on an ethylenediaminetetraacetic acid (EDTA)‐anticoagulated sample showed a sudden platelet drop to 80x10^3^/μL, prompting the suspicion of loss of response to the oral thrombopoietin receptor agonist. Review of the peripheral blood smear showed characteristic pictures of platelet satellitism (Figure 1,A–C). In approximately 30% of the involved polymorphonuclear neutrophils, platelet phagocytosis was also apparent (Figure 1,D–F). A spurious thrombocytopenia due to platelet satellitism was suspected, and a microscopic estimation of platelet count—considering even the platelets adhering to neutrophils' membrane or detectable within granulocytes' cytoplasm—yielded a result consistent with prior counts (120–140 x10^3^/μL). The occurrence of platelet satellitism was subsequently confirmed exclusively in blood slides prepared from EDTA‐ (Figure 1,G), and not from citrate‐ (Figure 1,H) or lithium heparin‐anticoagulated sample (Figure 1,I).

**FIGURE 1:**
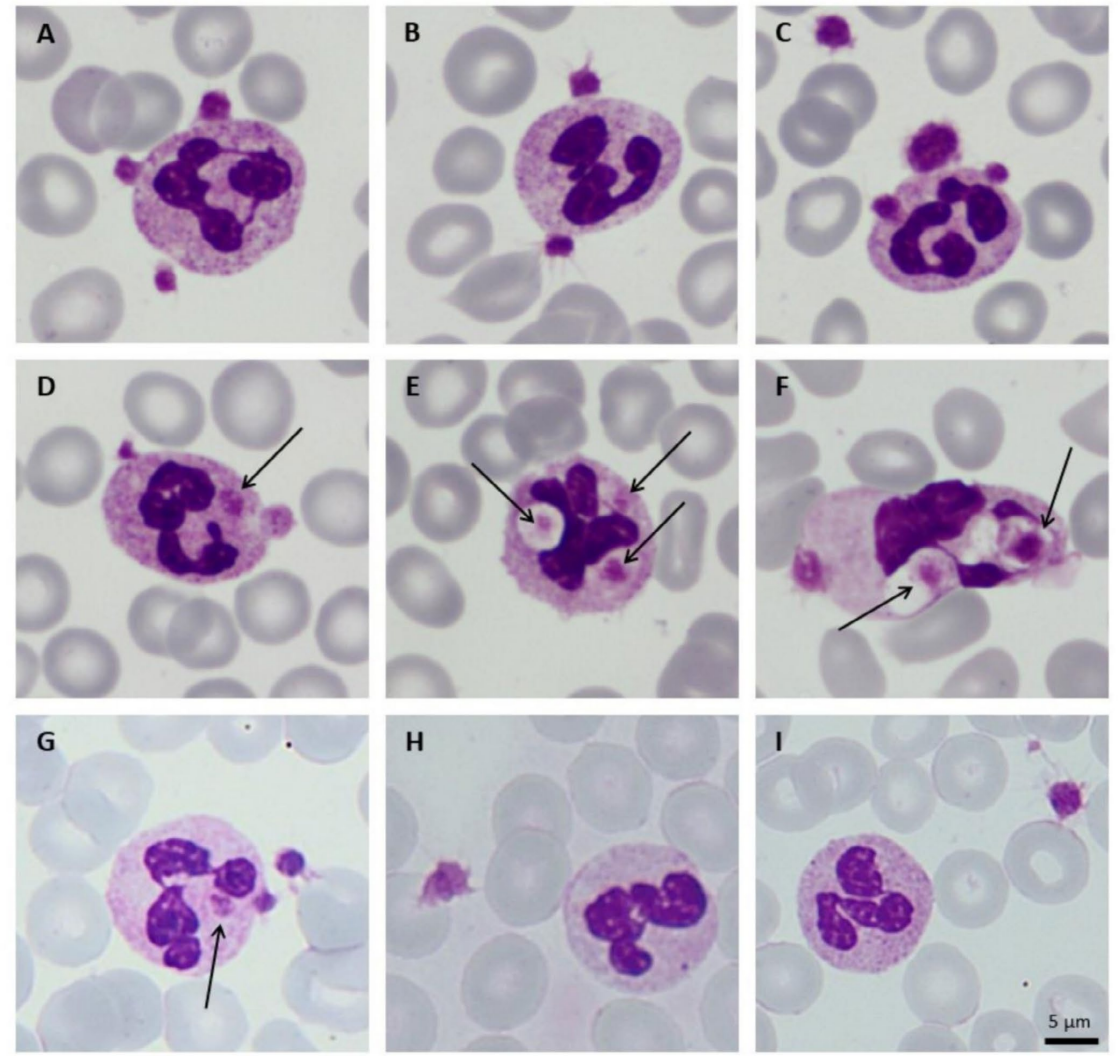
Light microscopy assessment of May‐Grunwald–Giemsa‐stained peripheral blood smears. Panel A–C show typical platelet satellitism with two‐to‐three platelets adhering to the membrane of polymorphonuclear granulocytes. Panel D–F display platelet satellitism with concomitant platelet phagocytosis (indicated by arrows). Panel G‐I confirms the occurrence of platelet satellitism with phagocytosis (arrow) exclusively in blood slides performed from an independent EDTA‐anticoagulated blood sample (G) and its absence in blood slides performed from citrate‐ (H) or lithium‐heparin‐anticoagulated blood (I). Scale bar corresponds to 5 μm.

Platelet satellitism is an EDTA‐associated in vitro phenomenon with much lower incidence than the “classical” EDTA‐dependent pseudothrombocytopenia, which causes false thrombocytopenia due to platelet agglutination. Its recognition still requires microscopic assessment of the blood smear [1, 2]. A noticeable spuriously reduced platelet count due to satellitism is exceptionally rare [3] but should be considered when there is an unexpected result, as illustrated in this case.

## Author Contributions

T.E., S.H., and L.B.K. collected clinical information. T.E. and C.Z. performed and interpreted blood films assessment by light microscopy. C.Z. wrote the manuscript. All authors helped with data interpretation, critically reviewed the manuscript, and approved the final version.

## Funding

The authors have nothing to report.

## Ethics Statement

The authors have nothing to report.

## Consent

The authors have nothing to report.

## Conflicts of Interest

The authors declare no conflicts of interest.

## Data Availability

The data that support the findings of this study are available from the corresponding author upon reasonable request.
